# Data of slurry quality, soil chemistry and crop yield and composition from a field experiment established 2011 comparing digested and undigested slurry

**DOI:** 10.1016/j.dib.2024.110201

**Published:** 2024-02-20

**Authors:** Tatiana F. Rittl, Anne de Boer, Reidun Pommeresche, Anne-Kristin Løes

**Affiliations:** a Norwegian Centre for Organic Agriculture (NORSØK), Norway; b Norwegian Institute of Bioeconomy Research (NIBIO), Norway

**Keywords:** Anaerobic digestion, Animal manure, Farm level biogas, Grass-clover ley, Organic management

## Abstract

The article presents relevant data from a long-term field experiment in Norway, comparing anaerobically digested and undigested slurry from organically managed dairy cows since 2011. Both the undigested and digested slurry originated from the same herd of cows and heifers. The dataset includes chemical analyses of slurry, soil characteristics at plot level of pH, extractable nutrients, and loss on ignition; crop yields, botanical composition (some years), and plant mineral composition (some years). These data supplement the findings presented and discussed in the research article *Anaerobic digestion of dairy cattle slurry – long-term effects on crop yields and chemical soil characteristics*[1].

Specifications Table

**Table:** 

Subject	Agricultural science
Specific subject area	Organic farming; organic fertilizers; soil properties, crop yield
Type of data	Excel files
How data were acquired	**Slurry** dry matter, pH and concentrations of macronutrients and ammonium-N (N min% of total N) were obtained from Eurofins or SINTEF Norlab, Namsos, Norway. **Soil chemical** analyses were performed by Eurofins; soil was sampled in 2011, 2013, 2016, 2018 and 2021. **Crop yields** were obtained annually for the grass system (2011-2021) and an arable system (2011-2013). The arable system was converted to grass in 2013. Fresh weight and dry weight were recorded. **Botanical composition** was measured some years in the grass system. **Plant mineral composition** was analysed some years.
Data format	Raw Calculated
Description of data collection	**Slurry** dry matter and chemical composition was measured in winter or spring for the batches of slurry to be applied in the field. **Soil samples** were taken from 0-20 cm soil layer with hand-augering. **Crop yields** from the ley were obtained from two cuts per season, usually in mid-June and mid-August. Arable crops were harvested by one cut for cereals and two cuts for ryegrass. Representative sub- samples were taken from the plant material from each harvest plot, weighed, then dried at 60°C for determination of the dry matter content. Parallel representative samples from the ley were taken for **botanical composition**: 0.5 kg of fresh plant material per plot was sorted into grass, clover and weeds, and the proportions of fresh and dry material was measured for each category. **Plant composition of** macro, micronutrients, and potentially toxic elements, elements of interest, fibres.
Data source location	Institution: Norwegian Centre for Organic Agriculture (NORSØK) City/Town/Region: Tingvoll/ Møre og Romsdal/ North-Western Norway Country: Norway Latitude and longitude (and GPS coordinates, if possible) for collected samples/data: 62°540’N,8°110’E.
Data accessibility	Repository name: Mendeley Data Data identification number: 10.17632/np8f797czv.1 Direct URL to data: https://data.mendeley.com/datasets/np8f797czv/1 Instructions for accessing these data: You are welcome to use this data e.g. to compare with your own data. To understand the data better, you are also very welcome to get in touch with the corresponding author.
Related research article	Rittl, et al., 2023. Anaerobic digestion of dairy cattle slurry – long-term effects on crop yields and chemical soil characteristics, Organic Agriculture, Volume 13, pages 547–563.

## 1 Value of the Data

• For anaerobic digestion (AD) of animal manure in farm-level biogas reactors, it is of high importance to assess how the AD affects manure quality as well as potential effect on soil characteristics and crop yields.
• Farmers considering investing in biogas treatment of animal manure, as well as their advisors, are the primary target group. Researchers studying AD and application of animal manure to field crops in general should also find the data relevant. The data can be used to understand the long-term effects of the application of digested and non-digested slurry on soil chemistry and crop yield. This is relevant, as such long-term experimental data are rare.
• The data include measurements from a long-term field experiment in Norway with application of digested and non-digested organic dairy cow slurry. Detailed information about soil chemistry is provided together with crop yield and botanical composition.

## 2 Data Description

In the dataset “Replication data for: Anaerobic digestion of animal manure – long-term effects on crop yields and soil characteristics” the following files were uploaded:

**Table:** 

01_ digested and undigested slurry
02_ soil chemistry
03_ yield
04_ botanical composition
05_ plant composition

The information available in « **01_ digested and undigested slurry**» excel file is:

**Table:** 

**Year**= year when the sampling was performed.
**Slurry type =**the type of slurry analyzed, anaerobically digested or non-digested.
**Container=** container which the sample was taken
**Date**= dates when the samples were taken.
**Dry matter**= dry matter content in %.
**pH (water) =** in slurry
**Total N=** g N in 1 kg^−1^ of slurry
**NH_4_-N =** g NH_4_-N in 1 kg of slurry
**NH_4_-N_tot=** % NH_4_-N of the total N
**P, K, Mg, Ca, S, Na =** g nutrient in 1 kg of dry slurry

* = missing data

The information available in «**02_ soil chemistry**» excel file is:

**Table:** 

**Year**= year when the sampling was performed, 2010, 2011, 2013, 2016, 2018 and 2021.
**Slurry**= the type of slurry applied in each plot, digested or undigested. None refers to the control plots where slurry was not applied.
**Rate**= slurry application rate in each plot. L= low; H= high
**Treatment code**= USH = undigested slurry high rate; USL = undigested slurry low rate; ADSH = digested slurry high rate; ADSL= digested slurry low rate; Control = no slurry application.
**Slurry N rate** = 110 and 220 kg N ha^−1^ yr^−1^ or 85 and 170 kg N ha^−1^ yr^−1^.
**Plot number** = from 1 to 40 (1-20 Grass system; 21-40 Arable System)
**Block** = from 1 to 8 (1-4 Grass system; 5-8 Arable System)
**Type of system** = grass or arable.
**Sampling day** = when the soil samples were taken, in dd.mm.yyyy.
**Soil depth**= the soil depth in which the samples were taken (cm).
**Laboratory soil density** = bulk density is measured by weighed cylindrical container having a known volume, which is filled by shock exposure with soil sample and then re-weighed for soil density calculation (kg/l air-dry sieved soil).
**pH=** in water**.**
**P-AL, K-AL, Mg-AL, Ca-AL, Na-AL=** mg nutrient extracted by ammonium acetate-lactate solution in 100 g^−1^ of air-dry sieved soil
**Loss of ignition =** % dry matter (DM)
**K-HNO_3_ =** mg K in 100 g^−1^ of air-dry sieved soil

* = missing data

The information available in « **03_ yield** » excel file is:

**Table:** 

**Year**= year when the sampling was performed.
**Slurry**= the type of slurry applied in each plot digested or undigested. None refers to control plot where slurry was not applied.
**Rate**= slurry application rate in each plot. L= low; H= high
**Treatment code**=USH = undigested slurry high rate; USL = undigested slurry low rate; ADSH = digested slurry high rate; ADSL= digested slurry low rate; Control = no slurry application.
**Slurry N rate (kg ha^−1^)** = 110 and 220 kg N ha^−1^ year ^−1^ or 85 and 170 kg N ha^−1^ year ^−1^
**Plot number** = from 1 to 40 (1-20 Grass system; 21-40 Arable System)
**Block** = from 1 to 8 (1-4 Grass system; 5-8 Arable System)
**Type of system**= grass or arable.
**Crop** = the type of crop each year: grass-clover, oats, ryegrass, spring wheat.
**Production year ley**= refers to the production year of the grass-clover ley
**Cut/Harvest** = 1 refers to the first or single cut/harvest, 2 refers to the second cut/harvest.
**Cut/Harvest day** = the day when each harvest was performed, in dd.mm.yyyy.
**Fresh weight**= weight of fresh biomass harvest in each plot, in kg m^−2^.
**Dry matter** = percentage (%) of dry matter (DM) in the harvested biomass.
**Dry weight** = weight of dry biomass harvest in each plot, in kg DM m^−2^.

* = missing data

The information available in « **04_ botanical composition**» excel file is:

**Table:** 

**Year**= year when the sampling was performed.
**Slurry**= the type of slurry applied in each plot digested or undigested. None refers to control plot where slurry was not applied.
**Rate**= slurry application rate in each plot. L= low; H= high
**Treatment code**= USH = undigested slurry high rate; USL = undigested slurry low rate; ADSH = digested slurry high rate; ADSL= digested slurry low rate; Control = no slurry application.
**Slurry N rate (kg ha^−1^)** = 110 and 220 kg N ha^−1^ year ^−1^ or 85 and 170 kg N ha^−1^ year ^−1^
**Plot number** = from 1 to 20.
**Block** = from 1 to 4.
**Type of system**= grass.
**Grass since**= the year when the grass was established in the experimental plots.
**Crop** = grass-clover, ryegrass, spring wheat, oats
**Harvest/Cut** = 1 refers to the first or single harvest, 2 refers to the second harvest.
**Harvest/Cut day** = the day when each harvest was performed, in dd.mm.yyyy.
**Clover**= the proportion of clover in a representative sample of the canopy, expressed on percentage on dry matter basis.
**Grass**= the proportion of grass in a representative sample of the canopy, expressed on percentage on dry matter basis.
**Weed**=the proportion of clover in a representative sample of the canopy, expressed on percentage on dry matter basis.

* = missing data

The information available in «**05_ plant composition**» excel file is:

**Table:** 

**Year=** year when the sampling was performed.
**Crop=** grass-clover, oats, ryegrass, spring wheat
**Slurry**=the type of slurry applied in each plot digested or undigested. None refers to control plot where slurry was not applied.
**Rate=**slurry application rate in each plot. L= low; H= high
**Treatment code**=USH = undigested slurry high rate; USL = undigested slurry low rate; ADSH = digested slurry high rate; ADSL= digested slurry low rate; Control = no slurry application.
**Slurry N rate** (kg ha^−1^) = 110 and 220 kg N ha^−1^ year^−1^ or 85 and 170 kg N ha^−1^ year ^−1^
**Plot number** = from 1 to 40, the numbers indicate which plots were combined for composite samples.
**Block =** from 1 to 8; (1-4 Grass system; 5-8 Arable System). It is the blocks combined for the composite sample.
**Cut/Harvest** = 1 refers to the first or single cut/harvest, 2 refers to the second cut/harvest.
**Lab**=laboratory where the analyses were performed

**Table:** 

## 3 Experimental Design, Materials and Methods

### 3.1 Experimental site

The long-term study was established in 2011 at Tingvoll research farm, North-Western Norway (62°54’N,8°11’E). Tingvoll research farm is owned by the Norwegian Centre of Organic Agriculture (NORSØK). Overall, the experimental soil had a medium to low status (< 4 mg 100 g^−1^ air-dry soil) of phosphorus (P) and very high status (> 120 mg 100 g^−1^ air-dry soil) of acid-soluble potassium (K). The soil texture is loamy sand, with about 11% of soil organic matter (SOM) [2].

### 3.2 Experimental design and management

A biogas plant was established at Tingvoll research farm in 2010, to produce biogas by anaerobic digestion of the slurry from the herd of 20-25 organically managed dairy cows. Concurrently, a long-term field experiment with 2 × 4 replicate blocks was established to study yields and soil characteristics by different manure application in a perennial grass-clover ley and an arable system. Low (L) and high (H) application levels of digested slurry (ADS) and untreated slurry (US) were compared, with a control with no slurry application. In the arable system, the high slurry level corresponded to about 170 kg total N ha^−1^ yr^−1^. This is equal to the maximal amount of N that may be applied annually per hectare from animal manure according to the nitrate directive. The low level was 50% of the high level and comprised 85 kg total N ha^−1^ yr^−1^. The arable system was only run during 2011-2013, and in 2014 the arable plots were converted to ley plots, maintaining the treatments ADSL, ADSH etc. with slurry rates adapted to the grass system. In the grass-clover ley, further called grass system, the low-level mimics an organically managed system purchasing about 30% of the energy intake for the cows as concentrates, amounting to 110 kg total N ha^−1^ yr^−1^, approximately 30 tons of slurry per ha and year. The high level is two times the low amount, 220 kg total N ha^−1^ yr^−1^. This level originally mimicked a conventional farm where mineral fertilizers were purchased in addition to concentrates, contributing to generally higher yield levels and larger amounts of manure available per hectare. The slurry was diluted by water to < 5% dry matter (DM) and applied by hand, using 10-liter cans.

Both the undigested slurry (US) and digested slurry (ADS) originated from the same herd of cows and heifers at Tingvoll research farm, except in 2011 when it was acquired from the Norwegian University of Life Sciences (NMBU). From 2011 to 2021, the number of organically managed cows in the herd on the research farm remained relatively stable, ranging from 20 to 25 individuals. The calving was always spread over the year, and feeding (proportion of concentrate, grazing etc.) was uniform over the years. Hence, the quality of the untreated slurry was quite similar over the period. Acquiring US and ADS from NMBU in 2011 had minimal impact on the chemical composition of the US as well as the ADS. At Tingvoll farm, both US and ADS were always collected simultaneously, ensuring that the ADS produced in the on-farm biogas plant was comparable with the US batch.

In the cow house at Tingvoll farm, the liquid manure floats to a collection pit where a pump makes the slurry circulate regularly. Another pump takes batches of this manure over to the nearby biogas plant. US was collected by an outlet on this slurry pipe. We aimed for mixing slurry batches from more than one date, but since the pump could not take small portions, this was not always possible. However, mixing of manure from several days is already achieved in the collection pit. Further, the feeding of the herd is quite stable because the calvings are evenly spread throughout the year. In winter and early spring when manure was sampled, the cows’ feed was silage conserved in round bales, and concentrates. ADS was collected via an outlet from the digester.

The biogas plant in Tingvoll is a conventional CSTR run at mesophilic conditions (37°C). It has been in operation since 2011. From 2011- 2018 the plant comprised of two horizontal CSTRs, each with an active volume of 30 m^3^. The current reactor is a standing CSTR with an active volume of 50 m^3^. The reactors have only been fed with dairy cow manure from the Tingvoll Farm. The residence time has typically varied between 25-30 days during the years of operation. The biogas from the plant is utilized in a Stirling engine with a maximum power output of 6 kW and a heat output of 30 kW.

Each batch of manure used in the experiment was stored in a closed 1 m^3^ tank, and chemical analysis (pH, Total N, NH_4_, P, K, Mg, Ca, S, Na) were performed of each tank at the laboratory of Eurofins or Nemko Norlab (Namsos, Norway). The reported levels of uncertainty were 10% for dry matter, and 20% for other analyses. Similar amounts of total N were applied with both types of slurry. By slurry application, the concentration of total N in each tank was used to calculate the exact amount of slurry to be applied on each experimental plot.

In 2016, the experiment was used to test whether addition of finely ground marble (calcium carbonate) to slurry could reduce N-emissions. For that, 2% (by weight) of ground calcium carbonate type T509 (97% CaCO_3_, about 150 mg sulphur (S) per kg DM, 90% <20 µm, d50= 4 µm) was added to digested and undigested slurry and applied in half of experiment (two blocks from the old arable system, two from the grass system) on May 10 and 11, 2016. The other half of the experiment) received the same amounts on June 16 after the first cut of grass-clover [3].

In 2018, the experiment was used for testing the application of struvite [4]. Half the plots (same selection of blocks as in 2016) received 40 kg P ha^−1^ as struvite in spring, whereas the other half received the similar amount of application after final harvest.

### 3.3 Crop types, sampling, and analyses

The crops in the arable system were oats (*Avena sativa L. var*.) in 2011, ryegrass (*Lolium perenne L. var.*) in 2012 and spring wheat (*Triticum aestivum L.*) in 2013. The wheat was a mixture of lines bred for organic farming purpose by Anders Borgen, Agrologica, Denmark. The ryegrass was lately sown after a non-successful seeding of forage rape and harvested twice. Oats were separated in grains and straw after drying in the laboratory. Spring wheat in 2013 was harvested before complete ripeness, and hence not separated in straw and grains.

The grass system was harvested twice every year, and the seed mixture was red clover (*Trifolium pratensis* L.), white clover (*Trifolium repens* L.), alsike clover (*T. hybridum*), timothy (*Phleum pratense* L), and meadow fescue (*Festuca pratense* L.), similar to what the farmers applies on the farmed fields. In 2014 and 2019, all experimental plots were ploughed together with the surrounding field, and the ley was re-established with green fodder as a cover crop. The green fodder was composed of peas (*Pisum sativum L*.), vetches (*Vicia sativa L.)* and oats. Since 2014, the arable system has been used for perennial ley as well, offering 8 replicate plots of each manure treatment ADSL, ADSH, USL, USH and Control.

In the arable system, annual weeds posed an increasing problem between 2011-2013 because the plot size did not allow for any mechanical regulation. This contributes to explain the missing effect of manure application on crop yield levels and that lower yields were obtained in 2012 and 2013 compared with 2011. The yield levels were generally very small when compared with the grass system and show how poor the plant production may become with a bad weed control, in spite of significant manure application.

To record the yields, a harvest plot was cut in the middle of the experimental plot, with a size of 1.2 x about 6.5. The exact length of each harvest plot was recorded. The total weight of fresh plant material was recorded, and a representative sample was dried to measure the dry matter yield.

From the freshly cut canopy, a representative sample of 0.5-1 kg plant material was taken and separated into grass, clover and weeds by hand. Fresh and dry weights of each fraction were determined to measure botanical composition of grass-clover ley.

Between 2011-2013, crop samples were sent Bioforsk for near infrared spectroscopy determination of Total N, macronutrients (P, K, Ca, S, Mg), FEm (energy content in the dry matter (DM)), PBV (protein balance in the rumen), and AAT (amino acids absorbed in the intestine). In 2014, samples of green fodder were sent to Eurofins to analyse macronutrients (P, K, S, Mg). In 2018, composite grass-clover samples were sent to Actlabs, Canada and analyzed for of Total N, macro (P, K, Ca, S, Mg), and micronutrients (Fe, Zn, B, Mn, Mo, Cu, Na) and Al. In 2021, twenty composite grass samples were sent to Nemko Norlab for determination of Total N, macro (P, K, Ca, S, Mg) and micronutrients (Fe, Zn, Co, B, Mn, Mo, Cu, Na, V), potentially toxic elements (Cd, hg, Pb, Ni, Sn, As, Se, Ag, Cr) and other elements of interest (Al, Si). Total N was determined by a modified Kjeldahl method. The concentrations of macro, micronutrients, and potential toxic elements were determined using ICP-MS [Internal method based on NS-EN ISO 17294- 2: 2016].

### 3.4 Soil chemistry

For analysis of plant nutrients and pH, topsoil samples (0-20 cm) were taken by hand-augering as composite samples from five locations from each side of each experimental plot, in total 10 augerings per plot with an inner diameter of 1.8 cm. Each sub-sample comprised ca. 45 g soil (fresh weight). The exact location of each sub-sample was measured by a ruler. For each location, the distance to the “long wall” of the plot (length 8 m) was 65 cm. The distance from the “short wall” of the plot (length 3 m) to the utmost locations was 2 m, and the distance between each location in between the utmost locations was 1 m. Samples were taken in spring 2011, 2013, 2016 and 2018 before slurry application. For extractable plant nutrients, standard soil analyses as used for assessment of soil fertility in Norway were carried out by Eurofins, Sweden. The reported levels of uncertainty were 20%. Before analysis, the soil was air dried and crushed to pass a 2 mm sieve (ISO 11464:2006). Soil pH was measured in a soil-water suspension (1:5 v/v, ISO 10390:2005). AL-extractable P, K, Mg and Ca were determined by the Swedish standard SS028310, which follows the method of Égnér et al. (1960), where the soil was extracted with a solution containing 0.1 M ammonium lactate and 0.4 M acetic acid, pH 3.75 in the ratio of soil to solution of 1:20 (w/v). The concentrations of elements were measured with ICP-AES (inductively coupled plasma atomic emission spectrometry). Concentrations of AL-extractable P, K, Mg and Ca in soil are abbreviated as P-AL, K-AL, Mg-AL and Ca-AL. Acid-soluble K was extracted by 1M nitric acid, HNO_3_ during heating, filtrated and analysed using ICP-AES (Pratt 1965, slightly modified). The concentrations of plant nutrients are presented as mg 100 g^−1^ air-dry sieved soil (2 mm). In the arable system, six samples had P-AL concentrations < 2 mg 100 g^−1^. To facilitate statistical analysis, these values were set to 1.5. Soil organic matter (OM) was measured by ignition loss at Eurofins, Sweden and is presented here as the percentage weight loss of oven-dry subsamples (∼10 g) of gravel-free fine earth (< 2 mm) after ignition in steel crucibles in a laboratory furnace at 500 +/- 10°C for 3 hours. The laboratory reports a 10% level of uncertainty for this analysis.

## CRediT author statement

**Tatiana Rittl:** Data curation, Conceptualization, Writing- Original draft preparation. **Anne de Boer**: Reviewing, responsible for field experiment management. **Reidun Pommeresche:** Reviewing. **Anne-Kristin Løes**: Establishing the experiment, Writing- Reviewing and Editing.

## Ethics Statement

The authors have read and follow the ethical requirements for publication in Data in Brief and confirming that the current work does not involve human subjects, animal experiments, or any data collected from social media platforms.

## Data Availability

Data from a long-term field experiment comparing digested and undigested cow slurry (Original data) (Mendeley Data).
